# Social support, health literacy, perceived control, and health-promoting behaviors in young and middle-aged lung cancer surgical patients: a structural equation model analysis

**DOI:** 10.3389/fpubh.2026.1767727

**Published:** 2026-05-14

**Authors:** Hui Yang, Yaxin Qiao, Saisai Liu, Yun Zhang, Keheng Wang, Jingru Chen, Ruiyun Chen

**Affiliations:** 1 Department of Nursing, Henan Provincial Intelligent Nursing and Transformation Engineering Research Center, Henan Provincial Key Medicine Laboratory of Nursing, Henan Provincial People's Hospital, Zhengzhou, Henan, China; 2 Zhengzhou University People's Hospital, Henan University People's Hospital, Zhengzhou, Henan, China; 3 Department of Thoracic Surgery, Henan Provincial Intelligent Nursing and Transformation Engineering Research Center, Henan Provincial Key Medicine Laboratory of Nursing, Henan Provincial People's Hospital, Zhengzhou, Henan, China; 4 Institute of Nursing and Health, Henan University, Kaifeng, Henan, China; 5 Institute of Nursing and Health, Zhengzhou University, Zhengzhou, Henan, China

**Keywords:** health-promoting behaviors, social support, health literacy, perceived control, lung cancer

## Abstract

**Background:**

Lung cancer is a significant public health challenge worldwide. For young and middle-aged patients undergoing lung cancer surgery, health-promoting behaviors offer a low-cost, high-impact approach for improving postoperative recovery and long-term health outcomes.

**Objective:**

This study explored the associations between perceived social support, health literacy, perceived control, and health-promoting behaviors in young and middle-aged patients undergoing lung cancer surgery.

**Methods:**

We employed a cross-sectional design to investigate 436 young and middle-aged patients undergoing lung cancer surgery who were recruited from three public hospitals between December 2024 and August 2025. Data were collected using the Sociodemographic Questionnaire, Perceived Social Support Scale, Chinese Health Literacy Management Scale, Cancer Experience and Efficacy Scale (cancer experience and control efficacy subscales), and Chinese Health-Promoting Lifestyle Profile-II. Descriptive analyses were conducted using SPSS 27.0, and AMOS 28.0 was used for structural equation modeling and mediation analysis.

**Results:**

Perceived social support, health literacy, and control efficacy were positively associated with health-promoting lifestyles, whereas cancer experience was negatively associated with health-promoting lifestyles. The structural equation modeling results indicated that the model fit was good (root mean square error of approximation = 0.063, *P* < 0.05). The mediation analyses indicated that health literacy, cancer experience, and control efficacy significantly mediated the relationship between perceived social support and health-promoting lifestyles.

**Conclusion:**

Perceived social support was significantly associated with healthy lifestyles among young and middle-aged patients undergoing lung cancer surgery. Health literacy, cancer experience, and control efficacy played mediating roles in this association. Early identification and interventions targeting these factors may enhance patients' adoption of health-promoting behaviors.

## 1 Introduction

Lung cancer represents a major global public health challenge, ranking first in incidence and mortality among malignant tumors worldwide (1). In recent years, the incidence of lung cancer among younger populations has shown a rising trend. In Asia, early-onset lung cancer cases among individuals under the age of 50 account for approximately 76% of the global total (2). In China, middle-aged and young patients with lung cancer accounted for approximately 42.4% of all lung cancer cases (3). The current treatment modalities for lung cancer include surgery, radiotherapy, chemotherapy, targeted therapy, and traditional Chinese medicine. Among these, surgery remains the primary therapeutic option for early- and mid-stage disease and is critical for improving patients' survival rate (4). Notably, the number of patients with lung cancer eligible for surgical intervention is projected to increase by approximately 60% between 2018 and 2040 (5). However, young and middle-aged patients undergoing lung cancer surgery face several physical, psychological, and social challenges, including complications resulting from postoperative lung tissue defects (6), difficulties in emotional regulation (7, 8), and restrictions in social functioning (9). In addition, middle-aged and younger patients are typically in pivotal stages of their career or academic development, bear their family's economic burden (10), or serve as primary caregivers and societal pillars. Upon diagnosis and surgical intervention, these individuals frequently experience significant disruptions in their capacity to fulfill these essential social roles. Compared with their older counterparts, young and middle-aged patients undergoing lung cancer surgery have longer life expectancies and, consequently, experience greater demands for long-term health management, functional recovery, and quality-of-life maintenance and a heightened reliance on health-promoting behaviors (HPB) (11).

During treatment, patients often grapple with guilt stemming from reduced social and family engagement alongside the psychological burden of disrupted personal aspirations and uncertainty regarding their long-term prognoses (12, 13). Additionally, patients with lung cancer are at risk for recurrence throughout the course of treatment (14). These issues significantly affect the quality of life and prognosis of patients after lung cancer surgery (15). According to the American Cancer Society, engaging in healthy behaviors can mitigate adverse symptoms, reduce recurrence and mortality risks among cancer survivors, and substantially improve health-related quality of life (16). In China, promoting healthy lifestyles among community residents has become the core goal of the Healthy China Initiative (17). Nevertheless, accumulating evidence indicates that patients with lung cancer frequently exhibit unhealthy behaviors including irregular physical activity (18), high dietary inflammation (19), and psychosocial maladjustment (13). Health-promoting behavior refers to a set of proactive multidimensional actions undertaken by individuals to enhance their overall health status. It encompasses six core dimensions: health responsibility, stress management, self-actualization, physical activity, interpersonal relationships, and nutritional practices (20). To date, most studies on HPB among patients undergoing lung cancer surgery have focused on isolated dimensions such as physical exercise (18), dietary and nutritional behaviors (19), and psychological stress management (13). This focus limits the understanding of HPB as a comprehensive and multidimensional construct. Furthermore, few studies examine the driving variables and their intrinsic relationships related to HPB in young and middle-aged patients undergoing lung cancer surgery based on scientific theories or models. Structural equation modeling (SEM) is a robust analytical approach that allows the integration of relationships among multiple variables and the examination of potential direct and indirect pathways linking these variables with HPB (21). Therefore, based on the health promotion model (HPM) (22), we constructed a structural equation model to explore the status of HPB among young and middle-aged patients undergoing lung cancer surgery and their relationships with relevant variables. In doing so, we aimed to provide evidence to inform the development of targeted clinical interventions.

## 2 Literature review and theoretical derivation

### 2.1 Social support and health-promoting behaviors

**Figure 1:**
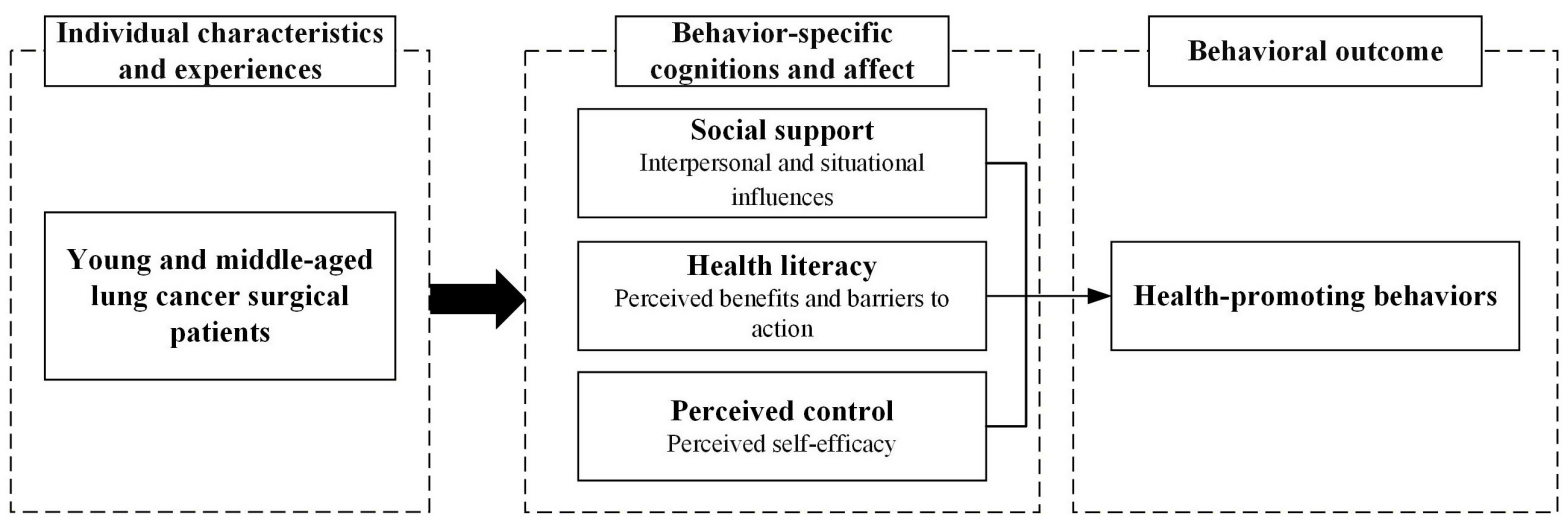
Theoretical framework of this study.

Health-promoting behaviors are shaped by several subjective and objective determinants. Pender's et al. (22) HPM provides a widely used theoretical framework for interpreting such behaviors. The model delineates three major categories of influencing factors: (1) individual characteristics and experiences, including prior related behaviors and personal factors; (2) behavior-specific cognitions and affect, encompassing perceived benefits and barriers to action, perceived self-efficacy, activity-related affect, and interpersonal and situational influences; and (3) behavioral outcomes, involving the commitment to a plan of action and the impact of immediate competing demands and preferences on the enactment of HPB. The theoretical framework of the study is presented in Figure 1.

From the perspective of the theoretical model's structure, social support is classified under “interpersonal and situational influences” in Pender's HPM. It is a core component of behavior-specific cognitions and affect and plays a significant predictive role in HPB. Social support refers to an individual's assessment of the assistance, emotional comfort, and information guidance received from family, peers, and community networks (23). In a specific population of patients undergoing lung cancer surgery, postoperative rehabilitation highly depends on social support networks such as family care and peer support. Recent studies have shown that social support can increase hope and advance recovery in patients with lung cancer (24). Both theoretical and empirical studies have shown that social support is a core exogenous variable that can affect factors such as self-efficacy, health literacy, and perceived control (25–27). Social support is directly related to health promotion behaviors (28–30) and plays a core role in the rehabilitation process of cancer patients (31). These findings emphasize that social support is a prerequisite for initiating and maintaining health promoting behaviors. Therefore, social support, as a core variable, has a solid theoretical foundation.

Accordingly, we proposed the following hypothesis:

*H1: Perceived social support is significantly associated with HPB in young and middle-aged patients undergoing lung cancer surgery*.

### 2.2 Mediating role of health literacy

Health literacy refers to an individual's ability to obtain, process, and understand essential health information and services to make informed health decisions (32). Previous studies have shown that higher levels of health literacy enable individuals to better recognize the potential benefits of health behaviors and to identify and overcome barriers encountered during their adoption and maintenance, thereby facilitating the development and sustainability of healthy behaviors (33, 34). This perspective is consistent with the core constructs of perceived benefits of action and perceived barriers to action in the Health Promotion Model. Accordingly, health literacy is widely regarded as an important cognitive resource influencing individual health behaviors. For patients with cancer, the processes of diagnosis and treatment are often accompanied by complex medical information and long-term self-management demands (35). To effectively cope with these challenges, patients need an adequate level of health literacy to understand disease-related information and make appropriate health decisions. Existing evidence indicates that health literacy is closely associated with health-promoting behaviors and health-related quality of life among cancer patients (36, 37). Furthermore, previous studies have suggested that health literacy may serve as a key mediator in the relationship between social support and health behaviors (38), indicating that higher levels of health literacy may facilitate patients' ability to translate external supportive resources into concrete health behaviors and healthier lifestyle practices.

Based on this, we proposed the following hypothesis:

*H2: Health literacy mediates the relationship between social support and HPB among young and middle-aged patients undergoing lung cancer surgery*.

### 2.3 Mediating role of perceived control

Perceived control refers to an individual's belief in their ability to draw on internal resources to influence external circumstances and achieve desired outcomes, and is conceptually closely related to self-efficacy theory (39). It is generally regarded as a multidimensional construct. Hou et al. developed the Cancer Experience and Control Efficacy Scale, which conceptualizes perceived control in terms of two related but distinct components: cancer experience and control efficacy. Cancer experience primarily reflects patients' cognitive and emotional appraisal of disease-related stress and burden, whereas control efficacy refers to their belief in their ability to cope with the illness and regulate their own behavior, emphasizing competence beliefs and behavioral motivation (40). In the cancer population, these two components may play different roles in relation to health behaviors. A more negative cancer experience may act as a hindering factor, as greater perceived illness burden can undermine engagement in beneficial health behaviors. By contrast, higher control efficacy may function as a facilitating factor by strengthening patients' motivation and confidence to adopt and maintain health-promoting behaviors (41, 42). Empirical studies further suggest that cancer experience and control efficacy may exert distinct directional effects (43).

Perceived control has been identified as an important correlate of health-promoting behaviors. Higher levels of perceived control have been associated with better self-care, lower psychological burden, improved adaptation to illness, and better quality of life (44–46). Including cancer experience and control efficacy as separate variables may therefore help clarify the distinct pathways through which perceived control is associated with health-promoting behaviors. Previous research suggests that perceived control may mediate the relationship between health literacy and health-promoting behaviors (36). Higher levels of health literacy may promote healthier lifestyles by improving patients' cancer-related experiences, such as reducing fear and misconceptions about the disease, and by strengthening control efficacy, such as increasing confidence in managing one's condition (36, 41). In addition, previous studies have shown that family care may improve perceived control among patients with breast cancer by alleviating negative illness experiences and enhancing disease control efficacy (43). Furthermore, when social support is low, lower perceived control has been associated with greater fear of cancer recurrence. In contrast, when social support is high, it may buffer the adverse effects of low perceived control on psychological outcomes (27). Taken together, these findings suggest that perceived control may be involved in the association between social support and health-promoting behaviors. By examining cancer experience and control efficacy as independent mediating variables, this study aims to further explore their associations with social support, health literacy, and health-promoting behaviors.

Based on this, we proposed the following hypothesis:

*H3: Perceived control (Cancer experience, control efficacy) mediates the relationship between social support and HPB in young and middle-aged patients undergoing lung cancer surgery*.
*H4: Health literacy and perceived control (Cancer experience, control efficacy) sequentially mediate the relationship between social support and HPB among young and middle-aged patients undergoing lung cancer surgery*.

**Figure 2:**
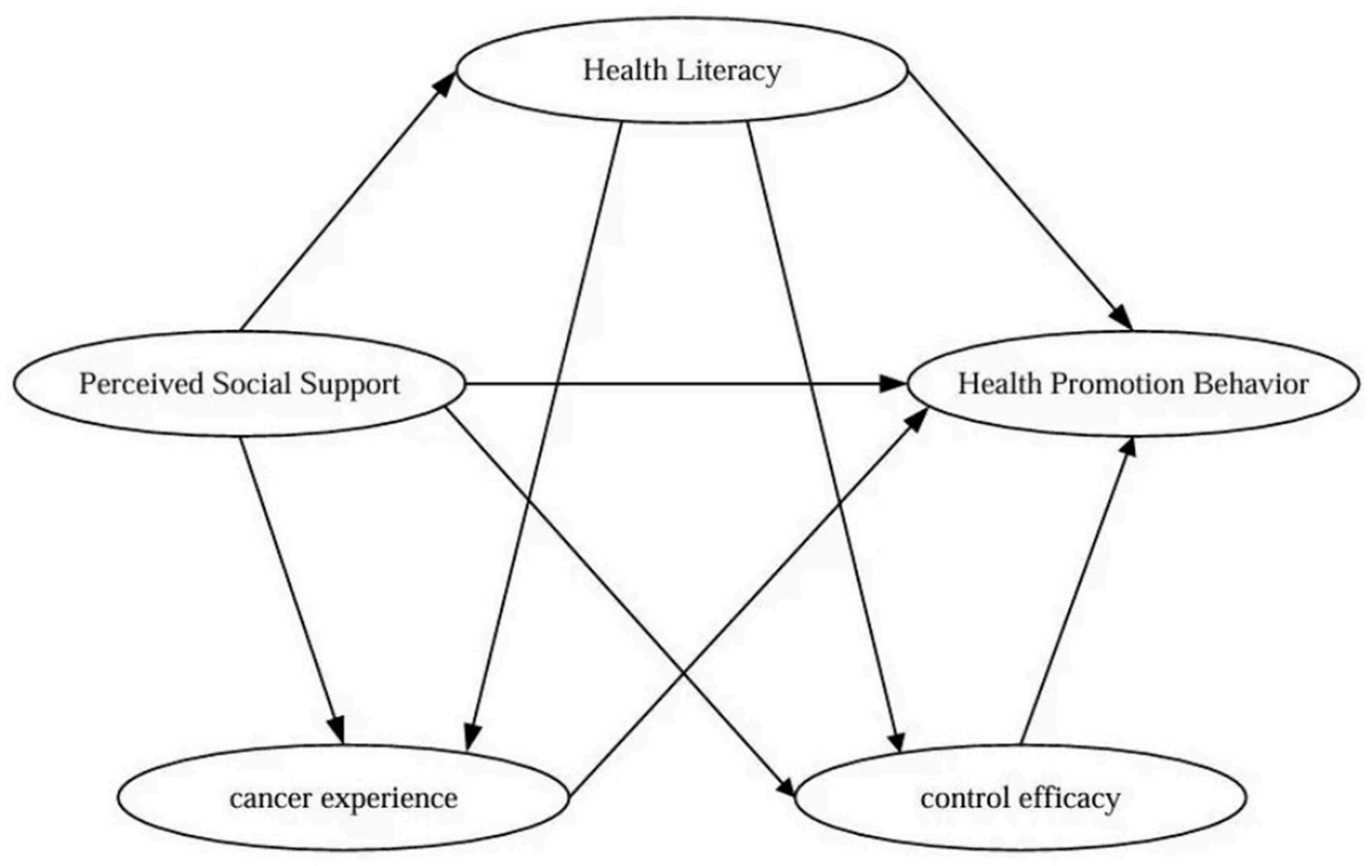
Hypothetical structural equation model of influencing factor paths in lung cancer health-promoting behavioral problem areas.

The theoretical model is shown in Figure 2.

### 2.4 Research objectives

Based on Pender's HPM and previous research, this study aimed to explore the association between social support, health literacy, perceived control, and HPB among young and middle-aged patients undergoing lung cancer surgery.

## 3 Methods

### 3.1 Study design

A cross-sectional survey was implemented to test the hypothesized relationships between health literacy, perceived social support, and perceived control within the conceptual model. This study followed the reporting guidelines of the Strengthening the Reporting of Observational Studies in Epidemiology (STROBE) statement (47).

### 3.2 Participants

Data were collected from December 2024 to August 2025, and all participants were recruited from three general public hospitals in Henan Province, China, using convenience sampling. The inclusion criteria were as follows: (1) meeting the diagnostic criteria for lung cancer outlined in the Clinical Diagnosis and Treatment Guidelines for Lung Cancer (2024 Edition) issued by the Chinese Medical Association (4) and undergoing surgical treatment; (2) aged 18–59 years; (3) being conscious, cognitively intact, and able to understand and complete the questionnaire; and (4) being fully informed of the study purpose and providing written informed consent. The exclusion criteria were as follows: (1) diagnosis of malignant tumors at sites other than the lung, (2) presence of distant metastasis or recurrent disease, and (3) a history of mental illness. The present study estimated a covariance-based structural equation model using IBM SPSS Amos (IBM Corp., Armonk, NY, USA). An *a priori* root mean square error of approximation (RMSEA)-based sample size analysis for overall SEM estimation was conducted in R using the semPower package. According to the Amos output, the model included 19 observed indicators, yielding 190 distinct sample moments, 51 free parameters, and 139 degrees of freedom. With α = 0.05, power = 0.80, and RMSEA = 0.05 as the target value, the minimum required sample size was estimated to be 136. The final sample of 436 therefore exceeded this requirement, indicating that the sample size was adequate for overall model estimation.

### 3.3 Data collection

Four researchers with systematic training administered a standardized questionnaire and collected data. Prior to data collection, the research team developed a unified protocol outlining the questionnaire distribution procedures and completion instructions. All investigators underwent standardized training to ensure consistency in survey administration. During the survey, the researchers explained the study objectives, content, and data confidentiality measures to the participants. The questionnaire was administered after obtaining written informed consent from the participants. During data collection, the researchers provided real-time support to and answered the questions raised by the participants. Two researchers checked, verified, and entered the data and removed invalid questionnaires to ensure data authenticity and completeness. The criteria for determining invalid questionnaires were as follows: (1) the presence of missing content, (2) responses showing obvious patterns, and (3) the existence of logical contradictions or inconsistencies with common sense.

### 3.4 Instruments

#### 3.4.1 Sociodemographic characteristics of participants

Demographic variables (sex, age, educational level, and marital status) were assessed using a self-developed questionnaire. Clinical variables (anatomical tumor location, histological subtype, and disease stage) were abstracted from electronic medical records.

#### 3.4.2 Health-promoting lifestyle profile II

Health-promoting behaviors were measured using the Chinese version of the Health-Promoting Lifestyle Profile-II, which was cross-culturally adapted by Cao et al. (48). It consists of 40 items classified into six domains: interpersonal relations (five items), health responsibility (11 items), stress management (five items), nutrition (six items), physical activity (eight items), and spiritual growth (five items). Responses to each item are scored on a scale ranging from “never” (one point) to “always” (four points). The total score ranges from 40 to 160, with higher scores indicating a stronger health-promoting lifestyle. In this study, the Cronbach's alpha values for each dimension and the total scale were 0.832, 0.928, 0.831, 0.874, 0.874, 0.907, and 0.965, respectively.

#### 3.4.3 Perceived social support scale

The Perceived Social Support Scale was developed by Zimet et al. (49), and the Chinese version was translated and revised by Zhong et al. (50). It consists of 12 items rated on a seven-point Likert scale across three dimensions: family support (four items), friend support (four items), and other support (four items). This scale is primarily used to assess the level of perceived social support, with higher scores indicating better perceived social support. In this study, the Cronbach's alpha values for each dimension and the total scale were 0.855, 0.884, 0.948, and 0.937, respectively.

#### 3.4.4 Health literacy management scale

The Health Literacy Management Scale (HeLMS) was developed by Jordan et al. (51). It is mainly used to measure patients' health literacy and comprehensively reflects the intrinsic meaning of their health literacy. We used the Chinese version of the HeLMS (52), which consists of four dimensions: information acquisition ability (nine items), economic support willingness (two items), communication and interaction ability (nine items), and willingness to improve health (four items), totaling 24 items. The scale is based on a five-point Likert scale, with scores ranging from “not at all” (one point) to “no difficulty” (four points). The higher the total HeLMS score, the higher the patient's health literacy level. In this study, the Cronbach's alpha values for each dimension and the total scale were 0.931, 0.917, 0.874, 0.842, and 0.945, respectively.

#### 3.4.5 Cancer experience and efficacy scale

The Cancer Experience and Efficacy Scale (CEES), developed by Hou (40), is primarily used to assess the perceived control of patients with cancer. It consists of two parts—cancer experience and control efficacy—comprising six dimensions and 29 items. The scale employs a five-point Likert scale, ranging from “strongly disagree” (one point) to “strongly agree” (five points), except for items 2 and 14, which are reverse-scored, while the remaining entries are positively scored.

The cancer experience subscale measures the physical, social, and emotional stress related to the disease, including personal strain (four items), emotional strain (six items), and socioeconomic strain (six items). The score ranges from 16 to 80 points; the higher the score, the more significant the patient's negative experience. In this study, the Cronbach's alpha values for each dimension and the total scale of cancer experience were 0.865, 0.910, 0.903, and 0.964, respectively.

The control efficacy subscale assesses patients' confidence in themselves, their family, and their healthcare team in managing their illness, including individual efficacy (five items), collective efficacy (five items), and proxy efficacy (three items). The score ranged from 13 to 65 points; the higher the score, the better the patient's response to the disease. The Cronbach's alpha values for each dimension and the total scale were 0.917, 0.958, 0.982, and 0.968, respectively.

### 3.5 Statistical analysis

Statistical analyses were performed using SPSS version 27.0 (IBM Corp., Armonk, NY, USA) and AMOS version 28.0 (IBM Corp., Armonk, NY, USA). Continuous variables were presented as mean ± standard deviation (SD), while categorical variables were expressed as frequencies and percentages. The normality of the observed variables was evaluated by examining the skewness and kurtosis values according to the criteria proposed by Kline (53). Data were considered approximately normally distributed when the absolute skewness was < 3 and the absolute kurtosis was < 8. Internal consistency reliability of the scales was assessed using Cronbach's α coefficient. Pearson's correlation analysis was conducted to examine the bivariate associations among the study variables. To examine the potential influence of common method bias, Harman's single-factor test was conducted using exploratory factor analysis. If the variance explained by the first unrotated factor was less than 40%, common method bias was considered unlikely to be a serious concern (54). We conducted SEM using AMOS 28.0 to examine the hypothesized relationships among the variables. This analysis consisted of two steps for a measurement model and structural model.

First, a confirmatory factor analysis was performed to test the measurement model and assess the relationships between the latent constructs and their observed indicators. Because the CEES, which measures perceived control, comprises two subscales (i.e., cancer experience and control efficacy), these were modeled as independent latent variables in our measurement model. The final measurement model included five first-order latent variables: perceived social support, health literacy, cancer experience, control efficacy, and health-promoting lifestyle. To improve model simplicity and the stability of parameter estimation, we adopted an item-packing strategy in which the total score of each dimension was used as the observation indicator of the corresponding latent variable (55, 56). Convergent validity was evaluated using standardized factor loadings, composite reliability (CR), and average variance extracted (AVE). When the AVE > 0.50 and CR > 0.70, convergent validity is considered acceptable (57, 58). The square root of the AVE for each latent variable should exceed its correlation with other latent variables to indicate good discriminant validity (59).

Second, the structural model was tested to evaluate the hypothesized relationships among the latent variables. Model fit was assessed using multiple goodness-of-fit indices, including the chi-square divided by degrees of freedom (CMIN/DF), root mean square error of approximation (RMSEA), goodness-of-fit index (GFI), adjusted goodness-of-fit index (AGFI), comparative fit index (CFI), normed fit index (NFI), relative fit index (RFI), incremental fit index (IFI), and standardized root mean square residual (SRMR). Good model fit is indicated by CMIN/DF < 3; RMSEA < 0.08; SRMR < 0.05; and GFI, AGFI, CFI, NFI, RFI, and IFI values greater than 0.90 (60). To test the indirect effects, a bias-corrected bootstrap method with 5,000 resamples was applied to estimate the 95% confidence intervals (CIs) for the mediation paths. An indirect effect is considered statistically significant if the 95% CI does not include zero. All statistical tests were two-tailed, and *P* < 0.05 was considered statistically significant.

## 4 Results

### 4.1 Sociodemographic and disease characteristics

Survey questionnaires were distributed to 450 potential participants, resulting in 436 valid responses and an effective response rate of 96.9%, which met the required sample size for the study. The sociodemographic and disease characteristics of the 436 participants are summarized in Table 1. Their mean age was 47.35 ± 9.73 years. The majority were female (*n* = 270, 61.90%), held a bachelor's degree or higher qualification (*n* = 115, 26.40%), were married (*n* = 413, 94.70%), and resided in urban areas (*n* = 246, 56.40%). Adenocarcinoma was the predominant histological type (*n* = 392, 89.90%). The cancer Tumor -Node -Metastasis (TNM) stages were as follows: stage I (*n* = 240, 55%), stage II (*n* = 156, 35.80%), and stage III (*n* = 40, 9.20%). Most patients (*n* = 362, 83%) were diagnosed within 12 months before recruitment.

**Table 1:** Sociodemographic and disease characteristics of the sample (*n* = 436).

Variable	Frequency (*n*)	Rate (%)
15.6-7.2,-1.3242ptAge (M ± SD; years)	47.35 ± 9.729	—
Gender		
Male	166	38.10
15.6-7.2,-1.3242ptFemale	270	61.90
Educational level		
Primary school and below	66	15.10
Junior high school	149	34.20
High school/vocational school	66	15.10
Junior college	40	9.20
15.6-7.2,-1.3242ptBachelor's degree or above	115	26.40
Marital status		
Single	17	3.90
Married	413	94.70
Divorce	5	1.10
15.6-7.2,-1.3242ptWidowed	1	0.20
Place of residence		
Urban areas	246	56.40
15.6-7.2,-1.3242ptRural areas	190	43.60
Medical expense payment methods		
Employee medical insurance	177	40.60
Urban and rural residents' medical insurance	241	55.30
15.6-7.2,-1.3242ptOther	18	4.10
Profession		
Farmer	114	26.10
Worker	38	8.70
Enterprise and institutional personnel	158	36.20
Unemployed	57	13.10
15.6-7.2,-1.3242ptOther	69	15.80
Primary caregiver		
Spouse	279	64.00
Child	115	26.40
Parents	24	5.50
Relative	13	3.00
15.6-7.2,-1.3242ptOther	5	1.10
Disease diagnosis time		
< 1 month	161	36.90
1–6 months	146	33.50
6–12 months	55	12.60
15.6-7.2,-1.3242pt>12 months	74	17.00
Disease understanding		
Don't understand	195	44.70
Partial understanding	201	46.10
15.6-7.2,-1.3242ptUnderstand	40	9.20
Tumor location		
Upper lobe of the left lung	106	24.30
Lower lobe of the left lung	97	22.20
Upper lobe of the right lung	94	21.60
Middle lobe of the right lung	74	17.00
Lower lobe of the right lung	60	13.80
15.6-7.2,-1.3242ptOther	5	1.10
Histological type		
Adenocarcinoma	392	89.90
15.6-7.2,-1.3242ptSquamous cell carcinoma	44	10.10
Disease stage		
Phase I	240	55.00
Phase II	156	35.80
Phase III	40	9.20

### 4.2 Common method bias test

Harman's single-factor test was conducted to assess common method bias. The results indicated that the variance explained by the first factor was 32.837%, which is below the critical threshold of 40.000%. Therefore, no significant common method bias existed.

### 4.3 Measurement model

The measurement model comprised five latent variables and 19 observed variables. First, the confirmatory factor analysis on the measurement model indicated that all factors had an AVE > 0.5 and CR > 0.7 (57, 58), demonstrating good convergent validity of the data (Table 2).

**Table 2:** Verification results of confirmatory factor analysis.

Latent variable	Dimension	Factor load (FL)	Average variance extracted (AVE)	Construct reliability (C.R)
PSSS	FAS	0.843	0.6791	0.8628
	FRS	0.903		
	OTS	0.715		
HeLMS	IAAD	0.831	0.5894	0.8514
	ESWD	0.738		
	CIAD	0.765		
	WTIH	0.733		
CEES-CE	PS	0.96	0.9101	0.9681
	ES	0.949		
	SS	0.953		
CEES-ES	IE	0.829	0.7892	0.9181
	CE	0.93		
	PE	0.903		
HPLP-II	IR	0.906	0.6833	0.9276
	HR	0.786		
	NT	0.871		
	PA	0.658		
	SM	0.875		
	SG	0.839		

PSSS, Perceived Social Support Scale; FAS, family support; FRS, friend support; OTS, other support; HeLMS, Health Literacy Management; IAAD, information acquisition ability; ESWD, economic support willingness; CIAD, communication and interaction ability; WITH, willingness to improve health; CEES-CE, The Cancer Experience and Efficacy Scale-Cancer Experience; PS, personal strain; SS, socioeconomic strain; ES, emotional strain; CEES-ES, The Cancer Experience and Efficacy Scale-Control Efficacy; IE, individual efficacy; CE, collective efficacy; PE, proxy efficacy; HPLP-II, Health-Promoting Lifestyle Profile II; IR, interpersonal relationships; HR, health responsibility; NT, nutrition; PA, physical activity; SM, stress management; SG, spiritual growth.

Second, multivariate normality was verified through SD, skewness, and kurtosis. We confirmed that the normal distribution conditions were satisfied (53). Therefore, each factor was normally distributed (Table 3). The scores on the Perceived Social Support Scale, HeLMS, CEES cancer experience subscale, CEES control efficacy subscale, and Health-Promoting Lifestyle Profile-II were 53.29 ± 12.288, 92.52 ± 15.250, 44.42 ± 12.567, 45.86 ± 10.593, and 106.58 ± 21.640, respectively. Based on Pearson's correlation analysis, HPB were significantly positively correlated with perceived social support, health literacy, and control efficacy (*r* = 0.483, *P* < 0.01; *r* = 0.559, *P* < 0.01; *r* = 0.502, *P* < 0.01, respectively) and negatively correlated with cancer experience (*r* = −0.557, *P* < 0.01; Table 4). The results of the discriminant validity test in the measurement model demonstrated that all correlation coefficients among the variables were lower than the square root of the AVE, indicating good discriminant validity of the model (Table 4).

**Table 3:** Descriptive statistics and distribution characteristics of the study scales and their dimensions (*n* = 436).

Variable	Scale/dimension score (mean ±SD)	Item mean score (mean ±SD)	Skewness	Kurtosis
**PSSS**	53.29 ± 12.288	4.44 ± 1.024	−0.032	−0.51
FAS	18.84 ± 4.117	4.71 ± 1.029	−0.308	0.076
FRS	17.78 ± 4.080	4.45 ± 1.020	−0.220	0.024
OTS	16.67 ± 5.772	4.17 ± 1.443	0.115	−0.823
**HeLMS**	92.52 ± 15.250	3.85 ± 0.635	−0.187	−0.402
IAAD	36.47 ± 6.750	4.05 ± 0.750	−0.782	0.493
ESWD	7.72 ± 1.778	3.86 ± 0.889	−0.313	−0.697
CIAD	33.12 ± 6.045	3.68 ± 0.672	0.210	−0.689
WTIH	15.21 ± 3.393	3.80 ± 0.848	−0.165	−0.938
**CEES-CE**	44.42 ± 12.567	2.78 ± 0.785	0.237	−0.511
PS	11.23 ± 3.285	2.81 ± 0.821	0.234	−0.572
ES	16.29 ± 4.859	2.72 ± 0.810	0.274	−0.346
SS	16.89 ± 4.814	2.82 ± 0.802	0.229	−0.468
**CEES-ES**	45.86 ± 10.593	3.53 ± 0.815	−0.964	0.128
IE	17.01 ± 3.786	3.40 ± 0.757	−0.425	−0.498
CE	17.74 ± 4.438	3.55 ± 0.888	−1.070	0.605
PE	11.11 ± 3.212	3.70 ± 1.071	−1.017	0.436
**HPLP-II**	106.58 ± 21.640	2.66 ± 0.541	−0.157	−0.159
IR	13.90 ± 2.597	2.78 ± 0.519	−0.308	−0.114
HR	27.97 ± 7.057	2.54 ± 0.642	0.288	−0.596
NT	17.62 ± 4.229	2.94 ± 0.705	−0.558	−0.141
PA	19.02 ± 5.395	2.38 ± 0.674	0.299	−0.505
SM	13.82 ± 2.933	2.76 ± 0.587	−0.116	−0.426
SG	14.25 ± 3.380	2.85 ± 0.676	−0.569	0.049

M, mean; SD, standard deviation. PSSS, Perceived Social Support Scale; HeLMS, Health Literacy Management; CEES-CE, The Cancer Experience and Efficacy Scale-Cancer Experience; CEES-ES, The Cancer Experience and Efficacy Scale-Control Efficacy; HPLP-II, Health-Promoting Lifestyle Profile II.

**Table 4:** The correlation and discriminative validity of variables (*n* = 436).

Latent variable	PSSS	HeLMS	CEES-CE	CEES-ES	HPLP-II
PSSS	**0.824**				
HeLMS	0.353^**^	**0.768**			
CEES-CE	−0.499^**^	−0.513^**^	**0.954**		
CEES-ES	0.571^**^	0.401^**^	−0.505^**^	**0.888**	
HPLP-II	0.483^**^	0.559^**^	−0.557^**^	0.502^**^	**0.827**

Diagonal bold numbers are AVE square root values, and numbers below diagonal are Pearson correlation coefficients (^**^P < 0.01). If the AVE square root value is greater than the correlation coefficients of the corresponding variable, it indicates good discriminative validity of the measured variable.

### 4.4 Structural equation model

**Figure 3:**
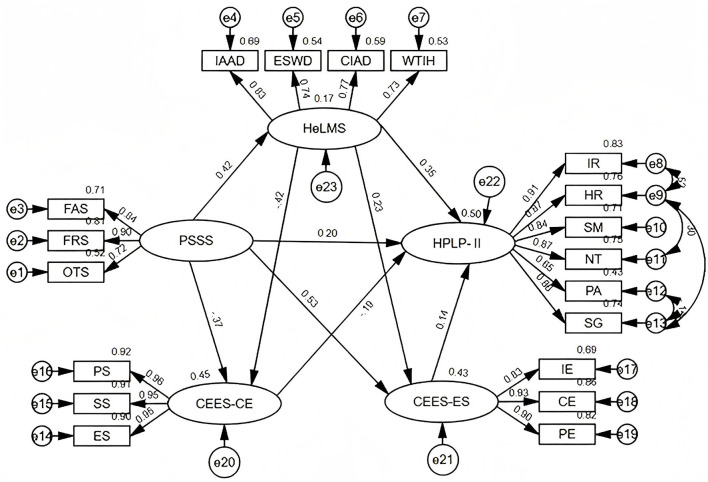
Structural equation model of health-promoting behaviors in patients undergoing lung cancer surgery. PSSS, Perceived Social Support Scale; FAS, family support; FRS, friend support; OTS, other support; HeLMS, Health Literacy Management; IAAD, information acquisition ability; ESWD, economic support willingness; CIAD, communication and interaction ability; WITH, willingness to improve health; CEES-CE, The Cancer Experience and Efficacy Scale-Cancer Experience; PS, personal strain; SS, socioeconomic strain; ES, emotional strain; CEES-ES, The Cancer Experience and Efficacy Scale-Control Efficacy; IE, individual efficacy; CE, collective efficacy; PE, proxy efficacy; HPLP-II, Health-Promoting Lifestyle Profile II; IR, interpersonal relationships; HR, health responsibility; NT, nutrition; PA, physical activity; SM, stress management; SG, spiritual growth.

The model fit results were as follows: CMIN/DF = 3.099, RMSEA = 0.069, CFI = 0.957, GFI = 0.895, and AGFI = 0.861. Because the initial model demonstrated an acceptable but suboptimal level of fit, it was refined based on modification indices using a stepwise modification approach. We introduced a limited number of error covariances among the dimensions of the health-promoting lifestyle construct. Residual correlations were allowed between health responsibility and spiritual growth, interpersonal relations and health responsibility, physical activity and spiritual growth, and health responsibility and nutrition. These modifications were theoretically justified as the dimensions of health-promoting lifestyles conceptually represented the interrelated components of health behavior. Previous studies have shown that individuals with a higher level of health responsibility are more likely to actively engage in multiple HPB and may experience greater spiritual growth in the process (61, 62). In addition, positive interpersonal relationships can enhance individuals' sense of responsibility for health management through mechanisms such as social support (63). Furthermore, physical activity is widely recognized as closely associated with psychological wellbeing and spiritual development (64). Therefore, the aforementioned modifications were considered theoretically reasonable. The final modified model demonstrated a satisfactory fit with the data: CMIN/DF = 2.733, RMSEA = 0.063, CFI = 0.965, GFI = 0.914, and AGFI = 0.882 (Table 5). The final standardized path map is shown in Figure 3.

**Table 5:** Model fitness results for structural equation modeling.

Fit index	Reference value	Initial value	Correction value
CMIN/DF	1–3 as excellent and 3–5 as good	3.099	2.733
GFI	>0.9 as excellent and >0.8 as good	0.895	0.914
CFI	>0.9 as excellent and >0.8 as good	0.957	0.965
AGFI	>0.9 as excellent and >0.8 as good	0.861	0.882
NFI	>0.9 as excellent and >0.8 as good	0.937	0.946
RFI	>0.9 as excellent and >0.8 as good	0.925	0.934
IFI	>0.9 as excellent and >0.8 as good	0.957	0.965
RMSEA	< 0.05 as excellent and < 0.08 as good	0.069	0.063
SRMR	< 0.05	0.055	0.049

CMIN/DF, chi-square degree of freedom ratio; GFI, goodness of fit index; CFI, comparative fit index; AGFI, adjusted goodness of fit index; NFI, normed fit index; RFI, relative fit index; IFI, incremental fit index; RMSEA, root mean square error of approximation; SRMR, standardized root mean square residual.

The parameter estimation for the modified health-promoting lifestyle model for patients undergoing lung cancer surgery is shown in Table 6. This study showed that nine pathways were statistically significant. Perceived social support explained 17.4% of the variance in health literacy. Perceived social support and health literacy together accounted for 45.1% of the variance in cancer experience and 42.8% of the variance in control efficacy. Moreover, perceived social support, health literacy, cancer experience, and control efficacy collectively accounted for 49.6% of the variance in health-promoting lifestyles.

**Table 6:** Parameter estimates for the structural equation model.

Paths	B	S.E.	β	C.R.	*P-*value	SMC
PSSS → HeLMS	0.563	0.076	0.417	7.398	< 0.001	0.174
PSSS → CEES-CE	−0.416	0.055	−0.375	−7.619	< 0.001	0.451
HeLMS → CEES-CE	−0.348	0.04	−0.423	−8.654	< 0.001	
PSSS → CEES-ES	0.398	0.043	0.526	9.322	< 0.001	0.428
HeLMS → CEES-ES	0.128	0.028	0.228	4.573	< 0.001	
PSSS → HPLP-II	0.114	0.033	0.200	3.501	< 0.001	0.496
HeLMS → HPLP-II	0.149	0.023	0.354	6.636	< 0.001	
CEES-CE → HPLP-II	−0.096	0.026	−0.188	−3.651	< 0.001	
CEES-ES → HPLP-II	0.104	0.039	0.138	2.677	0.007	

B, unstandardized estimate; S.E., standard error; β, standardized estimate; C.R., critical ratio; SMC, squared multiple correlation.

Table 7 presents the direct, indirect, and total effects of the examined variables on the health-promoting lifestyles of patients undergoing lung cancer surgery. Among them, health literacy had the strongest direct positive association with health-promoting lifestyles (β = 0.354). When indirect pathways were considered, perceived social support had the greatest overall association with health-promoting lifestyles (β = 0.537). The mediation analysis confirmed the mediating roles of health literacy, cancer experience, and control efficacy in the relationship between perceived social support and health-promoting lifestyles (Table 8). Five significant mediation pathways were identified: Path 1: perceived social support → health literacy → health-promoting lifestyles [β = 0.148, standard error (SE) = 0.029, 95% CI (0.100, 0.215)], accounting for 27.56% of the total effect; Path 2: perceived social support → cancer experience → health-promoting lifestyles [β = 0.070, SE = 0.023, 95% CI (0.030, 0.121)], accounting for 13.03% of the total effect. Path 3: perceived social support → control efficacy → health-promoting lifestyles [β = 0.073, SE = 0.032, 95% CI (0.016, 0.141)], accounting for 13.59% of the total effect; Path 4: perceived social support → health literacy → cancer experience → health-promoting lifestyles [β = 0.033, SE = 0.012, 95% CI (0.015, 0.063)], accounting for 6.15% of the total effect; Path 5: perceived social support → health literacy → control efficacy → health-promoting lifestyles [β = 0.013, SE = 0.006, 95% CI (0.003, 0.028)], accounting for 2.42% of the total effect. In summary, the results indicated that simple and chain-mediated pathways existed between perceived social support and health-promoting lifestyles. In the simple mediation pathway, health literacy, cancer experience, and control efficacy played independent mediating roles between perceived social support and health-promoting lifestyles. In the chain-mediated pathway, health literacy served as the initial mediating variable, sequentially associated with cancer experience or control efficacy and, subsequently, with health-promoting lifestyles.

**Table 7:** Standardized direct, indirect, and total effects.

Dependent variables	Independent variables	Direct effect	Indirect effect	Total effect
HPLP-II	PSSS	0.200	0.337	0.537
	HeLMS	0.354	0.111	0.465
	CEES-CE	−0.188	0.000	−0.188
	CEES-ES	0.138	0.000	0.138
HeLMS	PSSS	0.417	0.000	0.417
CEES-CE	PSSS	−0.375	−0.176	−0.551
	HeLMS	−0.423	0.000	−0.423
CEES-ES	PSSS	0.526	0.095	0.621
	HeLMS	0.228	0.000	0.228

**Table 8:** Testing the mediating effect of perceived social support on health-promoting lifestyles.

Paths	β	Proportion of the total effect (%)	Bootstrap SE	Bootstrap 95%CI		*P*-value
				Lower	Upper	
PSSS → HeLMS → HPLP-II	0.148	27.56	0.029	0.100	0.215	< 0.001
PSSS → CEES-CE → HPLP-II	0.070	13.03	0.023	0.030	0.121	0.001
PSSS → CEES-ES → HPLP-II	0.073	13.59	0.032	0.016	0.141	0.01
PSSS → HeLMS → CEES-CE → HPLP-II	0.033	6.15	0.012	0.015	0.063	< 0.001
PSSS → HeLMS → CEES-ES → HPLP-II	0.013	2.42	0.006	0.003	0.028	0.005

β, standardized estimate; bootstrap SE, bootstrap standard error.

## 5 Discussion

This study utilized the HPM as a theoretical framework to investigate the associations between social support, health literacy, perceived control, and HPB in young and middle-aged patients undergoing lung cancer surgery. The pathways identified in this study represent statistical associations rather than causal relationships.

Based on sex-specific case counts from GLOBOCAN 2022, women accounted for 36.6% of incident lung cancer cases worldwide and 37.9% in China (65, 66); thus, the female proportion in the present sample (61.9%) was higher than that observed in the general incident lung cancer population. This pattern may be partly explained by the rising incidence of lung adenocarcinoma among young and middle-aged non-smoking women in recent years (67). In addition, because participants were recruited from thoracic surgery wards, where 89.90% of patients were diagnosed with adenocarcinoma, the relatively high proportion of women in this study may reflect the evolving epidemiological characteristics of this specific clinical subgroup. Previous studies have suggested that lung cancer in women is associated with multiple factors, including genetic susceptibility, environmental exposures, and lifestyle-related factors such as cooking-related exposure (68–70). For example, exposure to ambient particulate pollution during women's reproductive years has been reported to be associated with an increased risk of lung cancer (68). In the Chinese sociocultural context, women often assume greater responsibility for household cooking, and long-term exposure to cooking fumes may therefore contribute to lung cancer risk (70, 71). In addition, higher participation in low-dose computed tomography screening among women may increase the detection of asymptomatic, early-stage lung adenocarcinoma, which may also have contributed to the higher proportion of women observed in the present sample (72). These demographic shifts highlight the importance of promoting healthy lifestyles in this growing patient subgroup. Nevertheless, this study was conducted in a single city in the Central Plains region of China, the results may have been influenced by regional characteristics. In addition, some eligible patients declined to participate during data collection, and selection bias therefore cannot be excluded.

The proposed structural equation model demonstrated a satisfactory overall fit. Participants' mean health-promoting lifestyle score was 106.58 (SD = 21.640), indicating a moderate level relative to the scale's midpoint (100.00). This aligns with the results reported by Zhou et al. (73) in patients with colorectal cancer. A potential explanation is that patients tend to prioritize immediate treatment outcomes over long-term behavioral modifications, which may weaken their health maintenance awareness and reduce their motivation to seek health information or adopt health-promoting practices. Moreover, participants' educational level was relatively low, with 64.4% having completed high school or vocational school or below. Additionally, 90.8% reported a partial or no understanding of their disease. This may prevent them from effectively processing health information and translating it into sustained HPB.

The structural equation model revealed a significant direct positive association between perceived social support and health-promoting lifestyles (β = 0.200, *P* < 0.001), consistent with prior evidence (74). This underscores the support of family, peers, and colleagues as a critical external resource that facilitate the adoption of healthy behaviors. Young and middle-aged patients undergoing lung cancer surgery who navigate multifaceted psychosocial challenges experience social support as a dual catalyst: it mitigates psychological distress and enhances mental wellbeing (29) while improving their quality of life, physical recovery, and treatment adherence (30). Within China's collectivist cultural context, social support has a heightened significance, with evidence indicating that multidimensional social integration expands networks to foster health promotion (75). Notably, the model identified three indirect pathways between perceived social support and health-promoting lifestyles, which are associated with health-promoting lifestyles through health literacy, cancer experience, and control efficacy. This indicates that social support operates not only through emotional or resource provision but also by enhancing patients' capacity to acquire and apply health knowledge and cultivate confidence in self-management. Consequently, strategically enhancing social support is pivotal to improving health-promoting lifestyles. Future initiatives should prioritize the development of multifaceted support networks that integrate the family, healthcare teams, peer groups, and community resources. Innovative approaches such as online support communities (76) and social prescribing (77) should be leveraged to establish personalized, stage-appropriate support systems aligned with individual patient needs throughout the oncology trajectory.

The SEM results showed that the direct association between health literacy and health-promoting lifestyles was the most significant (β = 0.354, *P* < 0.001). In addition, the mediation analysis indicated that the indirect pathway from social support to health-promoting lifestyles through health literacy accounted for the largest proportion of the total effect (27.56%). This finding underscores the important mediating role of health literacy in translating social support into health-promoting behaviors and is consistent with previous studies (38, 78). Various sources of social support may contribute to health literacy. Family support is important for appointment accompaniment, concern expression, and patient–provider communication (79), whereas support from friends, especially peers, may facilitate access to illness- and recovery-related information through experience sharing and information exchange (80). During disease management, middle-aged and young patients undergoing surgery for lung cancer often face a substantial symptom burden, a prolonged recovery trajectory, and challenges in information access and decision-making (81, 82). Effectively coping with these challenges depends largely on their ability to obtain, understand, appraise, communicate, and apply health-related information, namely, their level of health literacy (83). Accordingly, health literacy should be considered a key target for interventions aimed at improving health-promoting lifestyles in this population. In the present study, the mean total health literacy score was 92.52 ± 15.250, indicating a moderately high level of health literacy. Among the dimensions, the highest item mean score was observed for information acquisition ability, whereas communication and interaction ability showed a comparatively lower item mean score. This pattern may be related to several factors. First, although 64.4% of the participants had an educational attainment of high school/vocational school or below, the present study specifically focused on young and middle-aged patients undergoing lung cancer surgery, a group that may have relatively stronger learning adaptability and greater familiarity with accessing health information through multiple channels (84). Second, 70.4% of the participants had been diagnosed within the previous 6 months and were therefore in the early stage following diagnosis and surgical treatment. During this period, heightened perceptions of disease threat and stronger rehabilitation needs may have increased their attention to disease-related knowledge and health management information (85). However, the comparatively lower scores for communication and interaction ability may suggest limitations in information exchange, problem articulation, and patient–provider communication. Accordingly, future interventions should provide stratified health education according to patients' capacity to understand and use health information. Accessible, context-specific, and actionable educational materials, combined with structured checklists and the teach-back method, may help strengthen patients' ability to comprehend, express, and verify health-related information (86). In parallel, digital communication tools, such as interactive mobile applications or mini-programs, may also be considered to encourage patients to ask questions proactively and participate more actively in decision-making (87). In addition, intervention strategies should incorporate caregiver involvement, peer support, and healthcare provider-led communication support to better promote improvements in both health literacy and health-promoting lifestyles (88, 89).

This study examined two dimensions of perceived control, namely cancer experience and control efficacy. Cancer experience reflects patients' negative subjective feelings and emotional responses, such as fear and anxiety, that may accompany the course of the disease and shape their psychological adjustment. In contrast, control efficacy reflects patients' confidence in their ability to formulate and implement health-related behavioral plans, encompassing their self-perceptions and beliefs regarding their capacity to adopt positive health behaviors and effectively manage the disease (40). The results showed that cancer experience was negatively correlated with control efficacy (*r* = −0.505, *P* < 0.01). At the same time, discriminant validity testing supported the distinction between these two subscales, suggesting that they capture relevant yet independent aspects of perceived control. More importantly, they showed opposite associations with health-promoting behaviors: cancer experience was negatively correlated with health-promoting behaviors (*r* = −0.557, *P* < 0.01), whereas control efficacy was positively correlated with health-promoting behaviors (*r* = 0.502, *P* < 0.01). Examining these subscales separately may therefore facilitate a more nuanced understanding of their different roles in relation to health-promoting behaviors and improve the theoretical interpretability of the findings. Consistent with previous studies (90, 91), cancer experience was negatively correlated with perceived social support, health literacy, and healthy lifestyle behaviors. In other words, the more intense patients' negative cancer-related experiences were, the less likely they were to benefit from social support, understand and apply health-related knowledge, and engage in health-promoting lifestyles. This pattern is consistent with stress and coping theory (92). By contrast, control efficacy was positively correlated with these variables, suggesting that patients who felt more confident in their ability to plan and implement health behaviors were also more likely to utilize social support resources, apply health knowledge effectively, and engage in health-promoting behaviors, in line with Bandura's self-efficacy theory (93).

However, cancer experience and control efficacy should not be interpreted in isolation, as they may jointly shape patients' engagement in health-promoting behaviors. In the present model, the standardized path coefficient from perceived social support to control efficacy (β = 0.526) was numerically larger than the absolute value of that from perceived social support to cancer experience (β = −0.375), whereas the absolute value of the coefficient from health literacy to cancer experience (β = −0.423) was larger than that from health literacy to control efficacy (β = 0.228). These findings suggest that social support may be more closely related to strengthening behavioral confidence, whereas health literacy may be more closely associated with reducing illness-related uncertainty, fear, and helplessness. However, because no formal statistical tests were conducted to compare the magnitudes of these path coefficients, these observations should be interpreted as descriptive rather than inferential. From a clinical perspective, patients may present with varying combinations of characteristics, and these distinct profiles may influence the prioritization of intervention strategies. Patients with high cancer experience and low control efficacy may require priority attention to psychological distress while gradually strengthening self-management capacity through interventions such as fear-of-recurrence management, emotion regulation training, action planning, and self-efficacy enhancement. Patients with high cancer experience but high control efficacy may benefit more from approaches that combine emotional support with structured behavioral guidance. For patients with low cancer experience but low control efficacy, the main challenge may lie less in emotional burden than in insufficient confidence and reduced agency in translating knowledge into action; interventions for this group should therefore emphasize behavioral empowerment, task decomposition, and action planning. By contrast, patients with low cancer experience and high control efficacy may represent a relatively well-adjusted group, for whom the focus may shift toward maintaining healthy behaviors, reinforcing adherence, and providing ongoing informational support (94–96).

This study further identified two chained mediation pathways in the association between perceived social support and health-promoting lifestyles: perceived social support → health literacy → cancer experience → health-promoting lifestyles and perceived social support → health literacy → control efficacy → health-promoting lifestyles. These findings suggest that higher perceived social support was associated with higher health literacy, which was in turn associated with more positive cancer-related experiences or stronger control efficacy, and subsequently with higher levels of health-promoting lifestyles. However, the standardized indirect effects of these two serial mediation pathways were relatively small (0.033 and 0.013, respectively), accounting for only 6.15 and 2.42% of the total effect. This indicates that the independent contribution of these pathways to clinical intervention development may be limited. Nevertheless, the findings offer a supplementary perspective for understanding the psychological and cognitive factors that may underlie the association between perceived social support and health-promoting lifestyles in patients with cancer. Future research should employ larger samples and longitudinal designs to further clarify the temporal ordering and robustness of these mediating pathways, as well as their implications for practice.

### 5.1 Limitations

This study had several limitations. First, the sample was limited to young and middle-aged patients undergoing lung cancer surgery at three hospitals in the Henan Province, China. Moreover, a certain degree of selection bias may exist. Women were overrepresented in the final sample (61.9%) relative to the source population of surgical patients with lung cancer (43.17%), suggesting possible differential participation by sex. As data on non-participants were not systematically collected, the extent of this bias remains unclear, which may have reduced sample representativeness and limited the applicability of the findings. Future studies should consider expanding the sample size, patient demographics, and geographic scope to enhance the representativeness. Second, although SEM enables the examination of complex associations, it cannot overcome the inherent limitations of cross-sectional designs in establishing temporal relationships or causality. Longitudinal studies are warranted to investigate the trajectories of HPB and their determinants over time to provide evidence for stage-specific interventions. Third, the model did not account for socioeconomic and disease-specific characteristics, which may limit the reliability and generalizability of the results. Incorporating these variables in future research and conducting sensitivity analyses to assess their potential confounding effects will strengthen the robustness of our conclusions. Fourth, the reliance on self-reported data may introduce common method bias. Although the statistical tests indicated that the extent of common method bias was limited, it cannot be entirely excluded because of the inherent limitations of the study design. Future research should employ ecological momentary assessment techniques using smartphones to capture multiple real-time behavioral data points in context. Combining these techniques with objective clinical indicators could further enhance the precision and validity of behavioral assessments.

### 5.2 Implications for policy and practice

The findings of this study may have practical implications for promoting health-promoting behaviors among young and middle-aged patients undergoing lung cancer surgery. Based on the pathways identified in this study, differentiated intervention strategies may help improve the precision and effectiveness of nursing practice. First, health literacy accounted for the largest proportion of the mediating effect in this study (27.56% of the total effect). In addition, among its dimensions, “information acquisition ability” had the highest score, whereas “communication and interaction ability” had the lowest. These findings suggest that interventions should be tailored to patients' specific health literacy profiles. For patients with limited health literacy, simplified and easy-to-understand educational materials, particularly those using visual and graphic formats, may help reduce comprehension barriers and improve access to key information. Structured group education sessions incorporating interactive discussion and case sharing may further strengthen patients' understanding of disease-related knowledge and their ability to apply it in daily self-management. For patients with relatively high health literacy, greater involvement in shared decision-making, including participation through digital health platforms, may help capitalize on their autonomy and information-seeking capacity, thereby fostering more active engagement in disease management. Second, with respect to the cancer experience pathway, interventions may need to place greater emphasis on reducing negative illness-related experiences and psychological distress. Approaches such as emotion regulation training, action-planning support, and self-efficacy enhancement may help patients reframe negative disease experiences and engage more constructively with treatment and recovery. Third, with respect to the control efficacy pathway, interventions aimed at strengthening patients' sense of competence and behavioral agency may be particularly important. For example, phased goal-setting may help patients visualize progress more clearly, while timely feedback may reinforce their sense of achievement. In addition, peer-support or peer-guidance programs may further enhance control efficacy by providing social modeling, encouragement, and mutual motivation.

## 6 Conclusions

We constructed a structural equation model based on the HPM to explore the relationship between perceived social support, health literacy, cancer experience, perceived control, and health-promoting lifestyles in young and middle-aged patients undergoing lung cancer surgery. The study also examined the mediating pathways of perceived social support through health literacy, cancer experience, and control efficacy to health-promoting lifestyles. Our results provide valuable information for clinical practice. Future research should consider including more diverse populations from different regions, medical institutions, and professional backgrounds and employ longitudinal or interventional designs to validate the generalizability of these findings.

## Data Availability

The original contributions presented in the study are included in the article/supplementary material, further inquiries can be directed to the corresponding author.
